# Exogenous Magnesium Chloride Reduces the Activated Partial Thromboplastin Times of Lupus Anticoagulant-Positive Patients

**DOI:** 10.1371/journal.pone.0157835

**Published:** 2016-06-29

**Authors:** Takayoshi Tokutake, Hisami Baba, Yuji Shimada, Wataru Takeda, Keijiro Sato, Yuki Hiroshima, Takehiko Kirihara, Ikuo Shimizu, Hideyuki Nakazawa, Hikaru Kobayashi, Fumihiro Ishida

**Affiliations:** 1 Department of Clinical Laboratory, Nagano Red Cross Hospital, Nagano, Japan; 2 Department of Hematology, Nagano Red Cross Hospital, Nagano, Japan; 3 Center for Medical Education, Shinshu University School of Medicine, Matsumoto, Japan; 4 Department of Hematology, Shinshu University School of Medicine, Matsumoto, Japan; 5 Department of Biomedical Laboratory Sciences, School of Health Sciences, Shinshu University School of Medicine, Matsumoto, Japan; National Cerebral and Cardiovascular Center, JAPAN

## Abstract

The activated partial thromboplastin time (APTT) assay is a basic hemostatic assay based on the time it takes for clots to form in plasma samples after the addition of calcium chloride. It is used to screen for various coagulation disorders. Several previous reports have suggested that magnesium (Mg) might contribute to coagulation reactions by binding to specific coagulation proteins. We investigated the effects of Mg on the APTT. In healthy controls, the APTT was significantly prolonged in proportion to the increase in the concentration of magnesium chloride in the range from 2.1 to 16.7 mmol/L. Among eight samples from patients with various disorders that exhibited prolonged APTT, two samples demonstrated shorter APTT when Mg was added, both of which were from patients that were positive for lupus anticoagulant. When we examined 206 clinical APTT samples, we found that Mg shortened the APTT of two samples. These two samples were also from lupus anticoagulant-positive patients (*p*-value: <0.003). Our findings regarding the unique effects of exogenous Mg on the APTT of lupus anticoagulant-positive patients might shed light on the role of Mg in APTT assays and lead to the development of a novel screening method for lupus anticoagulant.

## Introduction

The activated partial thromboplastin time (APTT) assay is a basic and widely used hemostatic assay that is based on the time it takes for clots to form in plasma samples. It is used to screen for various coagulation disorders, especially those affecting the intrinsic and/or common coagulation pathways [1]. The APTT assay is composed of two steps 1) APTT reagents including phospholipids and a contact activator of citrate-anticoagulated plasma are added to the test sample, which is then incubated, and 2) the clotting time is measured after the addition of calcium chloride solution. Calcium ions, which are divalent cations, are a crucial component of the coagulation cascade. Magnesium ions, which are also divalent cations, are involved in numerous metabolic and biochemical reactions and are found in plasma at concentrations ranging from 0.7 to 1.0 mmol/L [2]. Magnesium ions are chelated with citrate when the APTT is measured. Previous reports have suggested that magnesium might contribute to coagulation reactions by binding to specific coagulation proteins, such as factor IX [3]. However, the effects of magnesium on the APTT have not been fully elucidated, although it has been reported to influence the prothrombin time [4].

We investigated the effects of magnesium on the APTT and found that the addition of magnesium shortened the APTT of lupus anticoagulant-positive patients.

## Materials and Methods

### Subjects

Patients with prolonged APTT were recruited. As controls, patients whose APTT were measured for various purposes and healthy subjects with normal APTT values were also included. The study protocol was approved by the institutional review boards (IRB) of Nagano Red Cross Hospital (approval number 52) and Shinshu University School of Medicine (approval number 3263) and was performed in accordance with the Declaration of Helsinki. Written informed consent for the blood collection was obtained from the subjects. Informed consent was waived in some cases when residual clinical samples were available, which was approved by both of the IRB.

### Detection of lupus anticoagulant

Lupus anticoagulant was detected according to the updated lupus anticoagulant detection guidelines of the International Society on Thrombosis and Haemostasis (ISTH)[5]. Briefly, the lupus anticoagulant screening was based on the prolongation of the APTT. When an inhibitor pattern was detected during a mixing test, the diluted Russell’s viper venom time and/or a phospholipid neutralization assay were utilized as confirmatory methods.

### Sample preparation

Venous blood was drawn into a collection tube containing 3.2% sodium citrate (VENOJECT II, Terumo, Tokyo, Japan) and centrifuged at 3,000 rpm for 10 minutes. Plasma was prepared and analyzed immediately or kept frozen at -80°C until it was analyzed.

### Effects of exogenous magnesium chloride on the APTT

For the APTT assay, we used a commercially available kit, the Thrombocheck APTT reagent (Sysmex, Kobe, Japan), which contains rabbit brain-derived phospholipids and ellagic acid, together with a Coapresta-2000 automated coagulation analyzer (Sekisui Medical, Tokyo, Japan). Based on the results of preliminary experiments, the calcium chloride concentration was fixed at 25.0 mmol/L (data not shown). Mixtures of 25.0 mmol/L CaCl_2_ and 6.3, 12.5, 25.0, or 50.0 mmol/L MgCl_2_ were prepared (Mg/Ca mix), resulting in final MgCl_2_ concentrations of 2.1, 4.2, 8.3, and 16.7 mmol/L, respectively. Briefly, after mixing 50 μl of the APTT reagent with 50μl of citrate-anticoagulated plasma and then incubating the mixture for two minutes at 37°C, 50μl of Mg/Ca mix were added, and the clotting time was measured.

For the screening examinations, the APTT was measured after the addition of 20.0 mmol/L CaCl_2_ with or without 25.0 mmol/L MgCl_2_, which gave a final MgCl_2_ concentration of 8.3 mmol/L. MgCl_2_ was considered to have shortened the APTT when the ratio of the APTT (seconds) obtained with MgCl_2_ to the APTT (seconds) obtained without MgCl_2_ was less than 1.00. This ratio was termed the Mg/Ca-APTT index.

### Statistical analysis

Comparisons between the groups were carried out using the Wilcoxon *t*-test, paired *t*-test, Mann-Whitney U-test, or Fisher’s exact test as appropriate.

## Results

**Fig 1:**
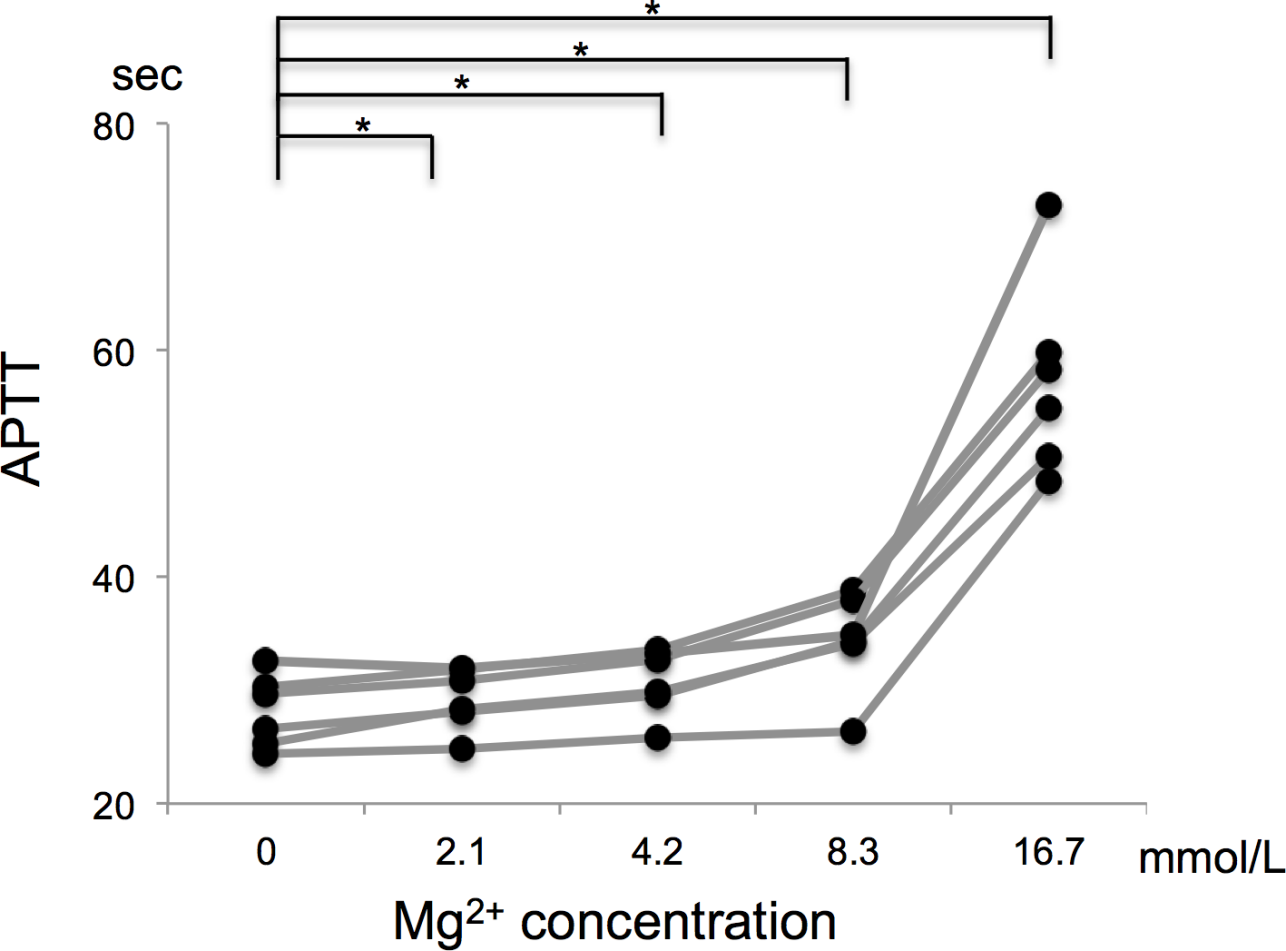
Effects of Mg^2+^ on the APTT of normal subjects. * indicates a *p*-value of <0.05

**Fig 2:**
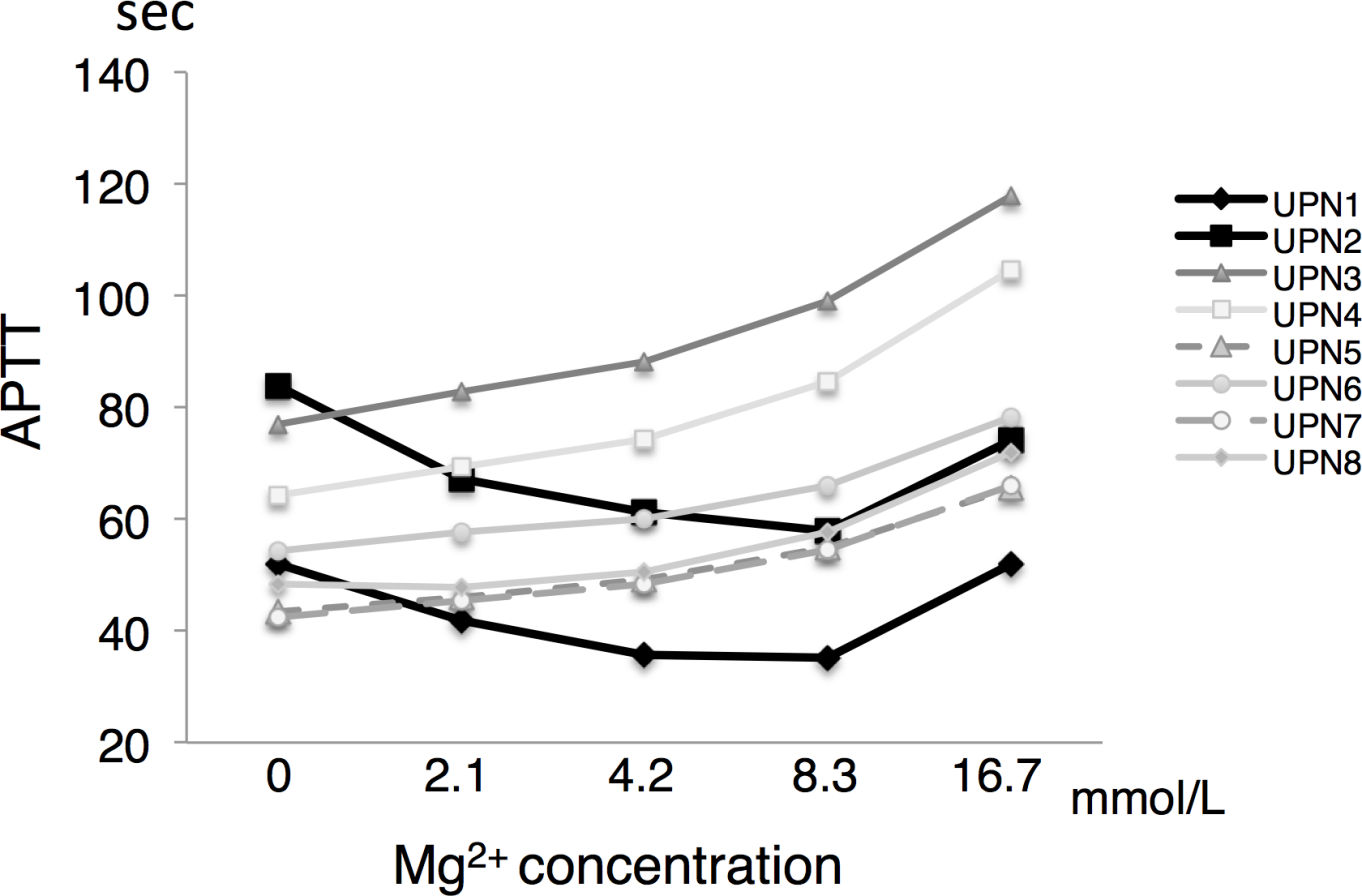
Effects of Mg^2+^ on the APTT of patients with prolonged APTT. The unique patient numbers (UPN) correspond to those shown in the table.

In an initial study of the healthy subjects, it was found that the APTT was shortest in the absence of MgCl_2_ and was significantly prolonged as the Mg^2+^ concentration increased (Fig 1). Next, we examined the effects of MgCl_2_ on the APTT of samples from patients with prolonged APTT. There were two and three patients that were positive for lupus anticoagulant and acquired anti-factor VIII inhibitors, respectively, and three patients with congenital coagulation factor deficiencies (Table 1). The congenital factor deficiencies included hemophilia A, hemophilia B, and factor V deficiency, and none of the patients with these conditions were positive for coagulation factor inhibitors. The APTT of two of the eight patients with prolonged APTT values shortened when MgCl_2_ was added during the APTT assay, while the APTT of the other six patients increased significantly when MgCl_2_ was added (Fig 2, unique patient numbers (UPN) 1 and 2). The coefficient of variation for the APTT assays performed with MgCl_2_ was 0.64% and 0.44% in a normal subject and UPN 1, respectively (n = 10 each). The addition of MgCl_2_ had similar effects on the APTT in both UPN 1 and 2, and the APTT-shortening effect of MgCl_2_ peaked at an MgCl_2_ concentration of 8.3 mmol/L (Fig 2). Both patients were positive for lupus anticoagulant (and one of them fulfilled the revised diagnostic criteria for antiphospholipid syndrome). In both patients, the APTT-shortening effect of magnesium ions was abrogated when the MgCl_2_ concentration was increased to 16.7 mmol/L (Fig 2).

**Table 1:** Characteristics of the patients with prolonged APTT.

UPN	Age (y.o.)	Sex	Diseases/examination findings	PT-INR	APTT(sec)
1	65	M	LA positive	1.01	52.0
2	69	F	LA positive/APS	1.27	83.8
3	83	F	acquired hemophilia	1.34	77.0
4	78	M	acquired hemophilia	1.25	64.3
5	79	M	acquired hemophilia	0.99	43.4
6	65	M	hemophilia A	1.09	54.3
7	41	M	hemophilia B	1.13	42.4
8	65	F	factor V deficiency	1.98	48.3

LA: lupus anticoagulant, APS: antiphospholipid syndrome

PT-INR: prothrombin time-international normalized ratio

APTT: activated partial thromboplastin time

In order to determine whether the APTT-shortening effects of exogenous MgCl_2_ are common, we examined 206 samples from cases in which the APTT assay was performed. The patients’ median age was 67 years (range: from one to 96 years). The coagulation tests were performed for various reasons including visits to the emergency department, preoperative or pre-delivery evaluation, and bleeding diathesis. Twenty-one of the 206 samples exhibited prolonged APTT. All 21 of these patients had received warfarin, heparin or rivaloxaban for various thrombotic disorders including myocardial infarction, cardiac failure, atrial fibrillation, and antiphospholipid syndrome. When the ratio of the APTT (seconds) obtained with MgCl_2_ to the APTT (seconds) obtained without MgCl_2_ (the Mg/Ca-APTT index) was less than 1.00, the sample was judged to be positive for MgCl_2_-induced APTT shortening. In total, 204 samples, including 19 samples from patients with prolonged APTT, were found to be negative for MgCl_2_-induced APTT shortening. The mean and standard deviation Mg/Ca-APTT index values of these patients were 1.33 and 0.17, respectively, and ranged from 1.08 to 2.38. Two of the 206 samples demonstrated shortened APTT after the addition of MgCl_2_. The Mg/Ca-APTT index values of these two patients were 0.75 and 0.83, respectively. Both of these patients had previously been shown to be positive for lupus anticoagulant, although no information about the presence/absence of lupus anticoagulant in the other 204 samples was available. Thus, MgCl_2_-induced APTT shortening was found to be significantly associated with lupus anticoagulant, but not with other disorders (*p*-value: <0.003). The relationship between the APTT recorded with and without MgCl_2_ is shown in S1 Fig. MgCl_2_ continued to have APTT-shortening effects on the two samples after they had been subjected to freezing and thawing (data not shown). The Mg/Ca-APTT index values of the 19 samples from the patients with prolonged APTT did not differ from those of the samples from the patients with normal APTT (*P* = 0.51).

## Discussion

Magnesium is capable of binding to the Gla domains of factors IXa and Xa in certain conditions [3, 6], which implies that magnesium ions might affect APTT assays. In the present study, the addition of magnesium prolonged the APTT of normal plasma at all of the examined concentrations, which is in contrast to the shortening effect it has on the prothrombin time, especially at low concentrations, such as those found in physiological conditions (data not shown). It remains unclear how magnesium affects *in vitro* coagulation assays. Interestingly, four samples, all of which were from lupus anticoagulant-positive patients, exhibited shorter APTT when Mg/Ca-mix was added during the APTT assay, whereas no such changes were seen in the other samples, including some from patients with other coagulation disorders. The mechanism through which lupus anticoagulant prolongs the APTT is not fully understood, but antiphospholipid antibodies in plasma are considered to interfere with phospholipid-dependent coagulation assays [7, 8]. Similarly, the mechanism through which magnesium shortens the APTT of lupus anticoagulant-positive patients and that by which magnesium prolongs the APTT of lupus anticoagulant-negative patients and normal subjects require further investigation. A previous study demonstrated that Mg^2+^ ions are able to replace Ca^2+^ ions at three of the eight Ca^2+^-binding sites within the factor IX Gla domain [6]. Furthermore, increases in Ca^2+^-binding affinity and significant alterations in Ca^2+^-binding cooperativity were detected in the presence of Mg^2+^ under physiological conditions [9]. In addition, it has been shown that Mg^2+^ ions potentiate synthetic phospholipid-dependent coagulation reactions involving factor IX, factor X, and prothrombin [10]. Almost all of these observations regarding divalent cations were obtained at physiological concentrations of calcium and magnesium, except in one report (Fig 1 in reference [11]). In the abovementioned studies, the effects of Mg^2+^ ions were calcium ion-dependent, which was consistent with our observation that MgCl_2_ barely induced any clot formation in the APTT assay (regardless of the concentration used) in the absence of Ca^2+^ (data not shown). Taken together with our findings that 1) the APTT was prolonged in a Mg^2+^ concentration-dependent manner in lupus anticoagulant-negative subjects and 2) APTT shortening was detected within a limited Mg^2+^ concentration range (between 2.1 mmol/L and 8.3 mmol/L) in lupus anticoagulant-positive patients, both of which were observed at highly elevated, non-physiological magnesium concentrations, it is assumed that magnesium might cause alterations in Gla domains or nearby structures that lupus anticoagulant antibodies bind to, which would prevent such antibodies from binding to their target and lead to the shortening of the APTT (in contrast to the APTT prolongation seen in the presence of physiological concentrations of magnesium, calcium, and phospholipids in APTT assays). Certain types of antiphospholipid antibodies also bind to coagulation-related proteins, such as prothrombin, in a phospholipid-dependent manner, and their role in APTT prolongation is unclear.

Although the number of patients examined in this study was quite limited, our findings regarding the APTT-shortening effects of magnesium in lupus anticoagulant-positive patients might aid the development of a novel screening method for lupus anticoagulant. In addition to studies of the effects of Mg^2+^ ions on lupus anticoagulant during APTT assays, examinations of their effects on other antiphospholipid antibodies and an evaluation of the diagnostic utility of an Mg^2+^-dependent APTT assay are currently underway.

## Supporting Information

S1 Fig Relationship between the APTT obtained with and without exogenous magnesium Ca-APTT: APTT obtained without exogenous magnesium, Mg/Ca-APTT: APTT obtained with 8.3 mmol/L exogenous magnesium, open circles: samples that exhibited APTT prolongation after the addition of magnesium (n = 210), closed circles: samples that demonstrated APTT shortening after the addition of magnesium and were also positive for lupus anticoagulant (n = 4). (TIFF)
