# Supplementary figures and images for: Exogenous Magnesium Chloride Reduces the Activated Partial Thromboplastin Times of Lupus Anticoagulant-Positive Patients
Source: PLoS One. 2016 Jun 29;11(6):e0157835. doi: 10.1371/journal.pone.0157835 (PMC4927146; doi:10.1371/journal.pone.0157835)

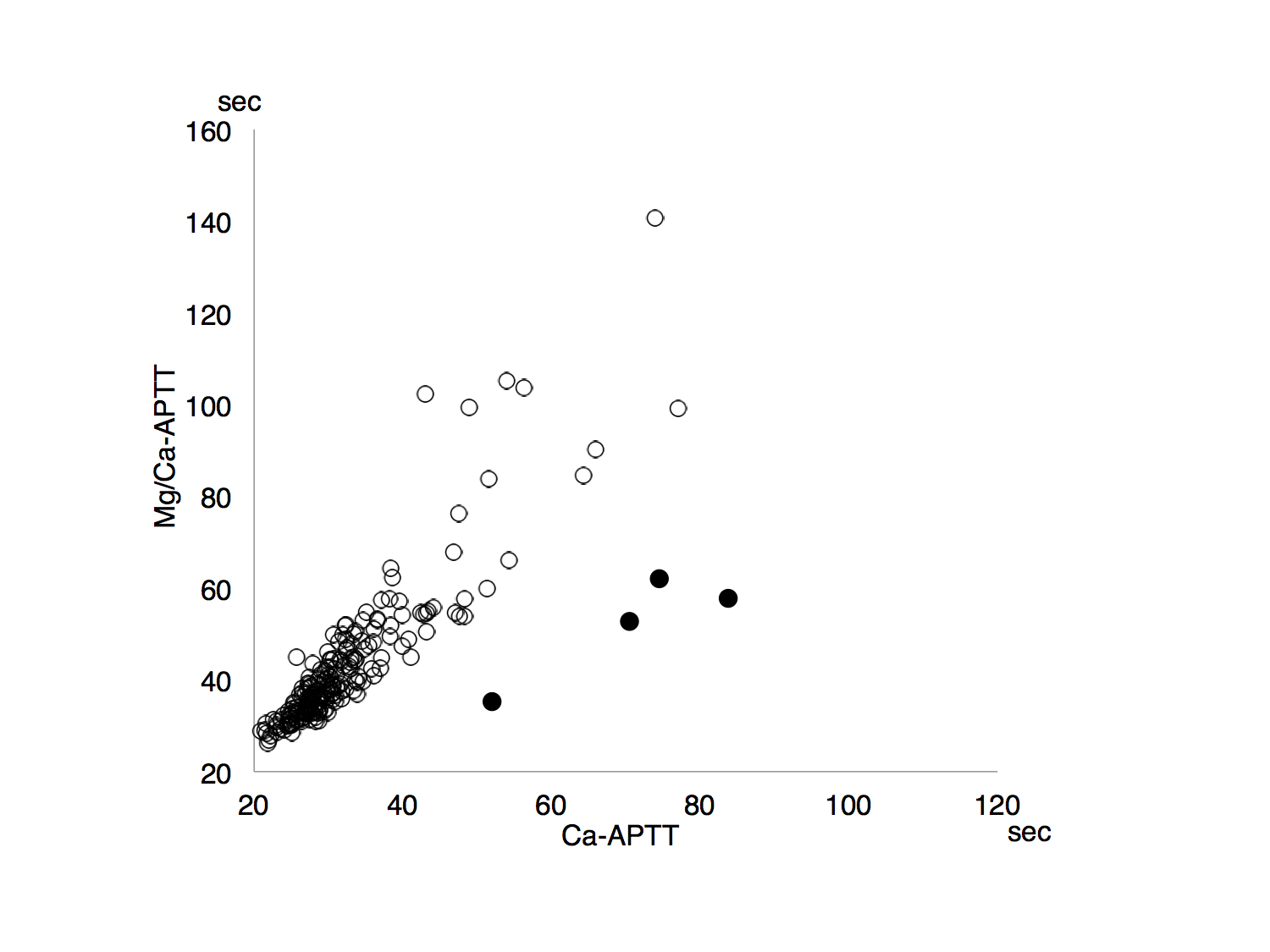

Supplement: S1 Fig — (TIFF) [file pone.0157835.s001.tiff]
